# The Impact of Culture in Weight Loss Intervention Effectiveness Among Hispanic American Women with Overweight or Obesity: A Systematic Scoping Review

**DOI:** 10.1177/15404153251383131

**Published:** 2025-10-17

**Authors:** Anna V. Garcia, Elizabeth Falls, Elizabeth Dodge, Basil H. Aboul-Enein

**Affiliations:** 1 College of Professional Studies, Applied Nutrition Graduate Program, 51686 University of New England, Portland, ME, USA; 2 College of Arts & Sciences, Health & Society Program, 14709 University of Massachusetts Dartmouth, North Dartmouth, MA, USA; 3 Faculty of Public Health and Policy, 156606 London School of Hygiene & Tropical Medicine, London, UK

**Keywords:** Hispanic women, overweight, obesity, cultural tailoring, intervention

## Abstract

**Introduction:** U.S Hispanic women bear a disproportionate burden of overweight and obesity. This review aims to examine and appraise the effectiveness of culturally tailored lifestyle modification interventions in this population. **Methods:** A review was conducted using seven databases for published studies between 2004 and July 2024 using PRISMA-ScR guidelines. The quality of studies was evaluated using the Effective Public Health Practice Project (EPHPP) tool. **Results:** Fourteen articles met criteria for inclusion. Interventions were delivered in-person in group and community settings, but varied in length, sample size, reporting of outcomes, and tailoring methods. All interventions were found to produce weight loss. Seven studies reported significant reductions in weight, eight in body mass index (BMI), and three in waist circumference. Across studies, the mean weight loss was −8.73 pounds, mean reduction in BMI units −1.85, and mean reduction in waist circumference −3.89 inches. Effective interventions included a bilingual and bicultural delivery, cultural modification of materials, incorporation of traditional Hispanic foods, and encouraged peer participation. **Conclusions:** Evidence demonstrates that culturally tailored lifestyle interventions are feasible, acceptable, and effective in producing weight loss and promoting healthier lifestyle practices among Hispanic American women. The findings of this review should be used to develop culturally relevant community programs and initiatives for the prevention and management of obesity among the population, providing rationale for further research to build upon the examined intervention strategies.

## Introduction

As we continue to see an alarming rise in overweight and obesity rates among Hispanic women living in the United States, prevention strategies continue to play a pivotal role towards addressing this growing public health issue. With many known adverse effects and risk factors, the burden of excess weight is detrimental on this rapidly growing population, of which 78.8% are affected by overweight or obesity today (U.S. Department of Health and Human Services Office of Minority Health, 2021). This places Hispanic American women at an increased risk for obesity and its comorbidities, including heart disease and stroke, type 2 diabetes, many types of cancer, and premature death (CDC, 2024).

Recent evidence demonstrates that overweight and obesity are more common among ethnic and racial minorities (Petersen et al., 2019). As the largest ethnic group in the U.S., Hispanic Americans—particularly women—are disproportionately more impacted (U.S. Department of Health and Human Services Office of Minority Health, 2021). Contradictorily, while Hispanic American women have a higher frequency of weight loss attempts as opposed to non-Hispanic white women (Marquez & Wing, 2013), they are 20% more likely to have excess weight (U.S. Department of Health and Human Services Office of Minority Health, 2021). Compared to the rest of the U.S. population, Hispanic Americans experience distinctive sociocultural factors that contribute to being less likely to follow physical activity guidelines and dietary recommendations (Marquez & Wing, 2013), which adds onto the complex set of challenges in the development and management of obesity in this group of individuals.

Because over 40% of Hispanic Americans were born in a foreign country, there are socioecological factors that may decrease the effectiveness of standard weight loss approaches among the Hispanic population living in the U.S. and may contribute to obesity rates in this population, including access to culturally appropriate foods, acculturation, cultural transition and assimilation (Evans et al., 2011). Literature suggests that immigrants arriving from Spanish-speaking countries are healthier than the American-born Hispanic population (Formagini et al., 2025; Lindberg et al., 2013; Ramírez et al., 2018). However, as they begin their lives in the U.S., the newly arrived often assume poor health behaviors resembling common American dietary habits characterized by large portion sizes, low nutritional quality, high caloric density, and high fat, sodium, and added sugar content (Pérez-Escamilla, 2011). Consequently, more acculturated Hispanic Americans tend to consume more added sugar, red meat, full-fat dairy, and have a lower consumption of legumes, rice, vegetables, and fruits (Evans et al., 2011).

Prior studies indicate that lifestyle modification-based weight loss interventions focusing on the general English-speaking population have been less successful among Hispanic American women (Morrill et al., 2021). Some of the reported challenges include poor adherence to diet, inability to keep up with required attendance, and not experiencing weight reduction (Marquez & Wing, 2013). While there is an evident desire to lose weight among the population, existing methods do not appear to be perceived as adequate. Therefore, the magnitude of the barriers that Hispanic American women experience with lifestyle behaviors expose a critical need for developing more effective evidence-based, culturally tailored programs designed specifically with these individuals in mind. For clarity, “culturally tailored programs” will be defined in this review as interventions that adapt to the commonly identified preferences and needs of Hispanic American women. Cultural tailoring consists of demographic and psychographic elements, which can include traditions, behavioral patterns, beliefs, attitudes, values, and religious views that pertain to a group of individuals with a shared cultural identity (Storey, 2022). In health-promoting lifestyle interventions geared toward Hispanic American women, cultural tailoring often considers dietary and cooking preferences, recipe modification, language, and music. As literature consistently shows, traditional non-tailored weight loss programs have failed to be effective for the Hispanic population (Lindberg et al., 2013). Considering how closely tied culture is to developed lifestyle habits, the role of cultural tailoring offers a compelling approach for weight management and health promotion in this population.

Despite the well-documented harmful consequences of obesity and the staggering rates at which underserved Hispanic American communities are at-risk for obesity and obesity-related comorbidities, they are overwhelmingly underrepresented in health, weight management, and obesity research. This review systematically assesses existing evidence related to culturally tailored lifestyle interventions and evaluate whether they are more successful in producing weight loss among Hispanic American women with overweight or obesity compared to standard, non-culturally tailored weight loss interventions. In doing so, the review explores the key challenges and nuances in weight management among this at-risk population for local communities, healthcare providers, and government agencies to consider and act upon.

The objective of this review is to determine whether culturally tailored lifestyle interventions can result in significant weight loss among Hispanic American women with overweight or obesity. To our knowledge, only one modern systematic review assessing the effectiveness of weight loss interventions in this population has been published to date (Morrill et al., 2021). However, no reviews evaluating the topic from a culturally tailored perspective exist. This review aims to fill this knowledge gap.

## Methods

### Search Strategy

We conducted a comprehensive literature search that included the following seven databases: PubMed, Cochrane, EMBASE, Scopus, Web of Science, PsycINFO, and Google Scholar. The search was performed for English-language articles published in the United States from year 2004 through July 2024 utilizing the Preferred Reporting Items for Systematic Reviews and Meta-Analyses (PRISMA) guidelines for scoping reviews (Tricco et al., 2018). A comprehensive list of key terms, words and phrases that were utilized for the search can be located the Supplementary Material. Only randomized controlled trials and studies with a quasi-experimental design measuring clinical outcomes in reduction of body weight, body mass index (BMI), or waist circumference were included. In addition, reference lists of included studies were manually searched for other articles that could be relevant for this review.

### Selection Criteria

Study inclusion criteria are summarized as follows:

- *Population:* adult women with overweight or obesity (BMI greater than 25, or a mean BMI greater than 30 among the sample) residing in the U.S., 18 years and older, self-identified as Hispanic or Latina
- *Interventions:* culturally tailored lifestyle interventions of greater than six weeks in duration, targeting diet and/or physical activity to reduce body weight, BMI, or waist circumference
- *Comparison:* Studies that measure change in weight, BMI, or waist circumference over time
- *Study outcomes:* measured change in weight (pounds or kilograms), BMI units, or waist circumference (inches or centimeters)
- *Study designs:* randomized controlled trials, quasi-experimental studies published between years 2004–2024

Studies were excluded if they recruited men, both men and women, or children or adolescents under 18 of age; included women who did not self-identify as Hispanic or Latina; did not apply cultural tailoring in an intervention; had a duration exceeding 24 months; included other racial or ethnic groups and were not exclusive to Hispanic American women; included participants with a BMI below 25 or a mean BMI below 30 among the sample; employed a different study design than a randomized control trial or a quasi-experimental design; were published prior to year 2004; assessed participants with a reported medical condition that can impact weight, participants who reported taking medicines or supplements that may alter weight, or pregnant participants; were published by the same author with similar studied outcomes; were published outside the U.S., or were not published in English.

Due to multiple terms often used interchangeably to describe this population, it should be noted that our search strategy included “Hispanic”, “Latina”, as well as subgroups with Latin American origins to represent individuals who identify as a person of Mexican, Cuban, South or Central American, Dominican, Puerto Rican, or other Spanish culture or origin. This has been a standard protocol with previous systematic reviews collecting data on Hispanic American populations (Morrill et al., 2021).

### Data Extraction

The primary researcher independently assessed articles that were identified for eligibility through a screening and inclusion process leading to a final selection of studies to be summarized in this review. During this process, the primary researcher removed duplicate articles and performed an initial screening of studies based on the title or abstract. From full-text articles being assessed for eligibility, the following data points were then extracted: participant and population characteristics, study design and setting, intervention characteristics, and study outcomes. Articles were assessed by study and intervention characteristics including cultural tailoring strategies, which are further examined in Tables 1 and 2. Study characteristics were classified into type of study, design, purpose, population, sample size, length and follow up, dropout rate, and Effective Public Health Practice Project (EPHPP) quality assessment score. Intervention characteristics were categorized into intervention type, implemented cultural tailoring strategies, delivery method and setting, measured target parameters and measurement frequency, and intervention outcomes.

**Table 1.** Characteristics of Included Studies.

Study	Design	Purpose	Population	Sample Size	Length/follow-Up	Dropout Rate	EPHPP Rating
Arredondo et al. (2017)	Cluster randomized controlled trial	To evaluate the impact of a faith-based intervention to promote physical activity in Latinas	Self-identified Hispanic American/Latina women aged between 18 and 65 years, inactive, frequent churchgoers, no BMI requirement for participation, mean post-intervention BMI of 30.39	436	24 months; no follow-ups	10%	Strong
Baldwin (2015)	Quasi-experimental (pre-test/post-test design); pilot study	To describe healthy lifestyle programs for underserved urban Hispanic neighborhood and report clinical outcomes and lessons learned	Self-identified Hispanic American/Latina women aged 35 years and older, English and Spanish speaking/reading; no BMI requirement for participation, mean baseline BMI of 38.7	48	4x 12-week programs across 2 years; no follow-ups	25%	Weak
Cherrington et al. (2015)	Quasi-experimental (pre-test/post-test design); pilot study	To develop and pilot test a theory-based, *promotora*-delivered, peer support weight loss intervention for Latina immigrants administered in a community setting	Self-identified Hispanic American/Latina women aged 18–65 with overweight or obesity (BMI ≥25), foreign-born, English-speaking/reading, 85.7% of Mexican descent	35	8 weeks with a follow-up at 6 months post-intervention	20%	Moderate
Faucher and Mobley (2010)	Randomized controlled trial; pilot study	To evaluate a culturally tailored portion control-focused intervention compared to standard care counseling on weight loss in low-income Mexican American women with excess weight	Self-identified Hispanic American/Latina women with overweight or obesity (BMI ≥25) and a desire to lose weight, 94.7% of Mexican descent	24	20 weeks, no follow-ups	40%	Moderate
Harralson et al. (2007)	Quasi-experimental (pre-test/post-test design)	To engage urban Latinas in physical activity and increase awareness of cardiac and metabolic risk factors	Self-identified Hispanic American/Latina women; 74% born in Puerto Rico, no participation eligibility criteria stated, mean baseline BMI of 31	225	12 weeks (year 1, 16 weeks (year 2); 3 post-intervention focus groups held across 2 years	48%	Weak
Hayashi et al. (2010)	Randomized controlled trial	To evaluate how effective a culturally appropriate lifestyle intervention was in improving health behaviors and cardiovascular risk profile	Self-identified Hispanic American/Latina women aged 40–64, low-income, under- or uninsured for healthcare coverage, at-risk for developing cardiovascular disease; no BMI requirement for participation, mean baseline BMI of 31.85	1093	12 months; no follow-ups	21%	Moderate
Keller and Cantue (2008)	Randomized controlled trial	To determine the effectiveness of walking on cardiovascular risk reduction in sedentary Mexican American women with obesity	Self-identified Hispanic American/Latina women (Mexican American), aged 45–70 years, postmenopausal, with obesity (BMI ≥30), sedentary	18	36 weeks; no follow-ups	52%	Weak
Koniak-Griffin et al. (2015)	Randomized controlled trial	To evaluate the effects of a culturally tailored lifestyle intervention on low-income immigrant Latinas with excess weight	Self-identified Hispanic American/Latina women, 35–64 years of age with overweight or obesity (BMI ≥25), low-income status, 83.9% of Mexican descent and low level of acculturation	223	6 months with a follow-up at 3 months post-intervention	13%	Strong
Lindberg et al. (2012)	Quasi-experimental (pre-test/post-test design); pilot study	To assess the feasibility of a culturally appropriate weight loss intervention to targeting Spanish-speaking U.S. Mexican women with obesity	Self-identified Hispanic American/Latina women (Mexican American), Spanish-speaking, 18–77 of age with obesity (BMI ≥30)	47	12 months, no follow-ups	55%	Moderate
Marquez and Wing (2013)	Randomized controlled trial; pilot study	To examine the feasibility and efficacy of a behavioral weight loss intervention adapted for Latinas	Self-identified Hispanic American/Latina women aged 18–65 with overweight or obesity (BMI of 27–50 kg/m^2^), English-speaking/reading, mostly foreign-born (66%) and of Caribbean descent	27	12 weeks with a follow-up at 12 weeks post-intervention	4%	Moderate
McCurley et al. (2017)	Quasi-experimental (pre-test/post-test design); pilot study	To test the effectiveness, feasibility, and acceptability of a peer-led, culturally appropriate lifestyle intervention for Latina women at-risk for type 2 diabetes by reducing risk through weight loss	Self-identified Hispanic American/Latina women with overweight/obesity (BMI ≥25) with no diabetes but at an elevated risk, 91.2% of Mexican descent	61	12 weeks with a follow-up at 12 weeks post-intervention	5%	Moderate
O’Brien et al. (2015)	Quasi-experimental (pre-test/post-test design); pilot study	To test the feasibility, acceptability, and effectiveness of a *promotora*-led diabetes prevention program in Hispanic American women	Foreign-born, self-identified Hispanic American/Latina women aged 20 or over, Spanish language fluency, with overweight or obesity (BMI ≥25), at-risk for diabetes	20	12 months; no follow-ups	10%	Moderate
Sanchez et al. (2021)	Quasi-experimental (pre-test/post-test design); pilot study	To assess the feasibility of implementing a *promotora*-led lifestyle intervention to improve obesity-related behaviors among Hispanic women residing in a predominantly Hispanic rural community	Self-identified Hispanic American/Latina women aged 18 and older responsible for the food shopping and food preparation in their homes, 70% born in Mexico; no BMI requirement for participation, mean baseline BMI of 30.2	49	5x 6-week programs across 1 year; no follow-ups	4%	Moderate
Seguin et al. (2019)	Quasi-experimental (pre-test/post-test design); pilot study	To assess the feasibility and efficacy of a culturally adapted lifestyle program with nutrition and physical activity components among a rural Latina population	Self-identified Hispanic American/Latina women with low acculturation, 40–70 years of age with overweight or obesity (BMI ≥25), sedentary or physically inactive, residing in a rural area	15	12 weeks with a focus group follow-up at 4 months post-intervention	27%	Moderate

**Table 2.** Characteristics of Interventions and Cultural Tailoring Strategies.

Study	Intervention	Cultural Tailoring Strategies	Delivery Method and Setting	Measured target Parameters	Frequency of Measurements	Intervention Outcomes
Arredondo et al. (2017)	24-month physical activity intervention with walking groups, cardio dance classes, and strength-training classes	Bilingual and bicultural staff Use of traditional foods in Latin cooking, images of Hispanic women in materials	Delivery in a community setting (Catholic churches frequented by Hispanic Americans) Group setting *Promotora*-led	Body weight* BMI* Waist circumference* Fitness level Physical activity Health conditions	Baseline, 12 months, 24 months	The culturally tailored intervention slightly decreased BMI in the intervention group (−0.1 kg/m^2^; p-values not reported). Although measured as part of the study, no tangible data was reported for changes in weight nor waist circumference. The intervention significantly increased physical activity.
Baldwin (2015)	Student-led 12-week lifestyle intervention targeting Hispanic American women by promoting a healthy diet and physical activity, and providing wellness education, cooking, and health coaching	Translation of materials into Spanish Public health clinical project in partnership with an urban Hispanic neighborhood	Delivery in a community setting Group setting Led by nursing students	Body weight (kg)* BMI* Waist circumference* HbA1c Blood pressure Physical activity Wellness health behaviors Risk factors for cardiovascular disease and diabetes	Baseline, 12 weeks	Participants’ BMI significantly decreased with a mean change of 3.22 BMI units (p < 0.05). Other significant improvements were observed in physical activity, diabetes and cardiovascular risk scores, and glycated hemoglobin (p < 0.05). No results nor tangible values were reported for changes in body weight, nor waist circumference, although measured as part of the study.
Cherrington et al. (2015)	8-week lifestyle intervention promoting healthy nutrition, physical activity, and diabetes prevention knowledge	Guided by a community advisory board Focus on family support	Delivery in a community setting Group setting *Promotora*-led	Body weight (kg)* BMI* Fasting glucose Lipid panel Dietary practices Physical activity Depressive symptoms	Baseline, 8 weeks, 6 months (follow-up)	There were encouraging results regarding the utility and efficacy of a culturally tailored weight loss intervention for Latina immigrants. The intervention resulted in statistically significant weight loss with mean body weight reduction of −2.1 kg (p < 0.001) and BMI reduction of 0.9 units (p < 0.001). Levels of physical activity and improvements dietary practices, and depressive symptoms increased significantly.
Faucher and Mobley (2010)	20-week culturally tailored healthy lifestyle-promoting dietary intervention focused on portion control	Bilingual and bicultural staff Food and recipes culturally specific to fit Mexican American cooking Economically sensitive, quick, low-cost meal options Focus on whole family's health Translated materials	Delivery in a community setting at a clinic providing women's health services Group setting *Promotora*-led	Body weight (lbs)* Physical activity	Baseline, 20 weeks	The study observed that a mean weight loss in the intervention group was greater at 6.57 lbs compared to a mean weight loss of 2.8 lbs in the standard care group. However, the difference between groups was not statistically significant (p = 0.47).
Harralson et al. (2007)	12-week (year 1) and 16-week (year 2) bilingual salsa dance aerobics exercise and nutrition education program	Bilingual and bicultural staff Translated materials Recipe modification with traditional Latin foods Salsa dancing for physical activity	Delivery in a community setting at a gym in a predominantly Puerto Rican neighborhood Group setting	Body weight (kg)* BMI* Waist circumference* Waist to hip ratio Blood pressure Depressive symptoms Health knowledge Perceived support and stress levels Medical conditions Self-rated health	Baseline, post-intervention (12/16 weeks)	The culturally tailored intervention resulted in decreases in BMI and abdominal obesity. Among completers, 50% of the women had a BMI over 30 indicating obesity, which decreased to 39% by the end of the program. The mean weight loss was approximately 2 pounds (p-value not reported). Waist circumference of >38 inches declined from 39% to 28%. Although measured as part of the study, no tangible values were reported for changes in BMI, nor waist circumference.
Hayashi et al. (2010)	12-month lifestyle intervention to improve dietary habits and physical activity	Culturally adjusted lifestyle counseling focusing on health behaviors Bilingual and bicultural staff Intervention offered in Spanish and English	Delivery in a community setting Group setting Led by community health workers (similar to *promotoras*)	Body weight (kg)* BMI* Total cholesterol & HDL Blood pressure Health-related behaviors	Baseline, 12 months	A culturally appropriate lifestyle intervention was associated with improvements in a small, yet statistically significant BMI reduction among the enhanced intervention group. Other improvements in health-related behaviors included systolic blood pressure levels and coronary heart disease risk assessment. No tangible values were reported for changes in body weight nor BMI, although measured as part of the study.
Keller and Cantue (2008)	36-week physical activity lifestyle intervention to promote walking among older Mexican American women	Nutritional education in dietary intake and healthier cooking methods Highlighted positive interpersonal relationships Reinforcement strategies through partners *(“comadres”, “gran amigas”)* for encouragement and support during physical activity	Delivered in a community setting (“*barrio*-delivered” across participants’ own neighborhoods that they considered familiar and safe) Group setting *Promotora*-led	Body weight (lbs)* BMI* Waist circumference* Body fat Adherence to physical activity	Baseline, 12 weeks, 36 weeks	Walking interventions favorably affected weight loss with significant reductions and main effect differences in body weight at post-intervention (−35.42 lbs in 3 days/week group; −22 lbs in 5 days/week group; p < 0.001) and BMI units (6.14 in 3 days/week group; 2.51 in 5 days/week group; p = 0.001.) No tangible values were reported for changes in waist circumference, although measured as part of the study.
Koniak-Griffin et al. (2015)	6-month culturally tailored lifestyle intervention to promote healthy diet and physical activity	Bilingual and bicultural staff Guided by a community advisory board Intervention offered in English and Spanish Culturally sensitive education materials	Delivery in a community setting, such as schools Intervention also included home visits Group setting *Promotora*-led	Body weight (lbs)* BMI* Waist circumference* Dietary behavior/habits Physical activity Blood pressure, blood lipids, glucose Health knowledge	Baseline, 6 months, 9 months (follow-up)	A culturally sensitive lifestyle intervention focusing on diet and physical activity significantly lowered participants’ body weight (p = 0.033), BMI (p = 0.046), and waist circumference (p = 0.038). At program completion, the intervention group experienced a mean reduction of −1.46 lbs in weight (−2.25 lbs at follow-up), −0.33 (−0.41 at follow-up) in BMI units, and −1.53 cm (−2.99 cm at follow-up) in waist circumference.
Lindberg et al. (2012)	12-month culturally tailored behavioral obesity care weight loss program focused on diet and physical activity	Intervention in Spanish Bilingual and bicultural staff Minimal materials; available in English and Spanish Emphasis on group activities Focus on Mexican traditions and beliefs Exercise options included salsa dancing	Delivery in a community setting at a local healthcare clinic Group setting Led by female clinicians and *promotoras*	Body weight (kg)* BMI* Dietary intake Physical activity	Baseline, 6 months, 12 months	A culturally sensitive behavioral lifestyle intervention is feasible to develop and implement to treat obesity. Statically significant mean post-intervention weight loss was −7.2 kg (p < 0.0001), and mean reduction in BMI was −5.5 units (p < 0.0001).
Marquez and Wing (2013)	12-week culturally tailored behavioral weight loss program promoting lifestyle change in diet and exercise	Bilingual and bicultural staff Guided by a community advisory board Materials providing nutritional content of traditional Latin foods Materials with modified recipes of traditional Latin dishes Culturally adapted nutrition education Focus on celebrating traditional meals through adjusted cooking methods	Delivery in a community setting Group setting Led by female interventionists (similar to *promotoras*)	Body weight (kg)* BMI* Weight loss self-efficacy Exercise self-efficacy Family social support for exercise	Baseline, 12 weeks, 24 weeks (follow-up)	A behavioral weight loss intervention targeting Latinas is feasible. In two culturally adapted intervention groups, significant weight loss (−4.7 kg and −4.3 kg; p < 0.01) was achieved at post-intervention; follow-up (−5 kg and −4.7 kg; p < 0.01). Nearly half of participants lost at least 5% of their baseline body weight.
McCurley et al. (2017)	12-week cultural adaptation of a lifestyle curriculum emphasizing health and lifestyle issues through portion size modeling, cooking demonstrations, and group exercise	Bilingual and bicultural staff Covered Latin cultural elements (foods, ethnicity-related risk factors, cultural beliefs) Inclusion of peers and family into lifestyle changes Childcare provided to facilitate adherence	Delivery in a community setting Group setting *Promotora*-led	Body weight (lbs)* BMI* Waist circumference (inches)* Blood pressure Depressive symptoms Perceived stress Perceived barriers to healthy eating and physical activity	Baseline, 3 months, 6 months (follow-up)	At post-intervention, participants achieved a mean reduction of −7 lbs, representing 4.1% body weight reduction, and at follow-up, an additional −6.6 lb weight reduction was observed (p = 0.28). Mean BMI reduced by 1.5 units at post-intervention, (p = 0.27) and waist circumference by 1.7 inches (p = 0.37), however, these changes were not statistically significant. Significant improvements were observed for dietary behaviors, stress, and depression symptoms.
O’Brien et al. (2015)	12-month lifestyle intervention to promote healthy eating and physical activity for weight loss and reduction of diabetes risk	Bilingual materials Targeted messages about healthy behavior change in Latin families Culturally appropriate tools for dietary self-monitoring	Delivery in a community setting by an organization serving Hispanic Americans Group setting *Promotora*-led	Body weight (lbs)* BMI* Waist circumference* Blood pressure and plasma glucose Insulin levels Hemoglobin A1C Lipids	Baseline, 12 months	A culturally tailored intervention demonstrated feasibility, acceptability, and effectiveness in Hispanic American women. At post-intervention, participants achieved a statistically significant mean reduction of BMI units (−1.9; p < 0.001), waist circumference (−4.3 cm; p < 0.001), and body weight (−10.8 lbs; p < 0.001), corresponding to −5.6% of baseline weight. 42% of participants achieved at least 7% weight loss. Significant reductions in blood pressure, LDL cholesterol, and insulin levels were also observed.
Sanchez et al. (2021)	6-week culturally tailored, interactive workshop series focusing on low-cost, easy cooking of healthy food and physical activity	Female staff Delivery in English and Spanish Developed using low-literacy principles	Delivery in a community setting Group setting *Promotora*-led	Body weight (lbs)* BMI* Self-perceived weight Physical activity Dietary habits	Baseline, 6 weeks	Delivering culturally tailored workshops is a feasible approach to promoting healthier lifestyle practices among Hispanic women, and it can help increase health awareness in their families. 54.3% of participants had a decrease in body weight ranging from 0.2 to 11.4 lbs. Mean BMI change was 0.1 units. No statistically significant changes in weight nor BMI were observed/expected by researchers given the short intervention duration.
Seguin et al. (2019)	12-week culturally tailored nutrition and physical activity program to promote healthy diet and physical activity, and to reduce obesity-promoting habits	Female interventionists Bilingual and bicultural staff Guided by a community advisory board Intervention options in English and Spanish Translated materials Program highlighted the role of family Focus on common social and economic barriers Guidance on culturally relevant foods and recipes Exercise options included Latin dancing	Delivery in a rural community setting by a community organization serving Hispanic American women Group setting Led by community health educators of a local nonprofit (similar to *promotoras*)	Body weight (kg)* BMI* Waist circumference* Cardiorespiratory fitness Physical activity Dietary behavior/habits Self-efficacy for diet and physical activity	Baseline, 12 weeks	A culturally sensitive lifestyle intervention focusing on diet and physical activity produced significant changes in body weight (−1.5 kg; p = 0.009), BMI (−0.6; p = 0.005), and waist circumference (−3.0 cm; p = 0.008). Post-intervention focus group revealed that participants found the culturally adjusted intervention successful, motivating and enjoyable.

For target outcomes (changes in body weight, BMI, or waist circumference), unless mean differences were provided in the original study, they were calculated by the primary researcher. Given the variations and nuances across the studies, and per Cochrane systematic review protocol and guidelines (Deeks et al., 2019), a meta-analysis could not be completed due to the heterogeneity of populations, study designs, differences in reporting, and intervention focus points.

### Quality Assessment

To assess the level of quality for each selected study, we utilized the EPHPP quality assessment tool. The EPHPP is a validated and reliable instrument to evaluate both randomized controlled trials and quasi-experimental studies, and its use is appropriate for systematic reviews of effectiveness (Armijo-Olivo et al., 2012; Thomas et al., 2004). With this tool, the quality of each primary research article was evaluated in terms of selection bias, study design, confounders, blinding, data collection, and attrition rate. Based on these components, the primary and secondary authors assessed the quality of each selected study by rating them as “weak”, “moderate”, or “strong”. Discrepancies were resolved by a third author as needed.

## Results

### Screening and Inclusion

**Figure 1.**
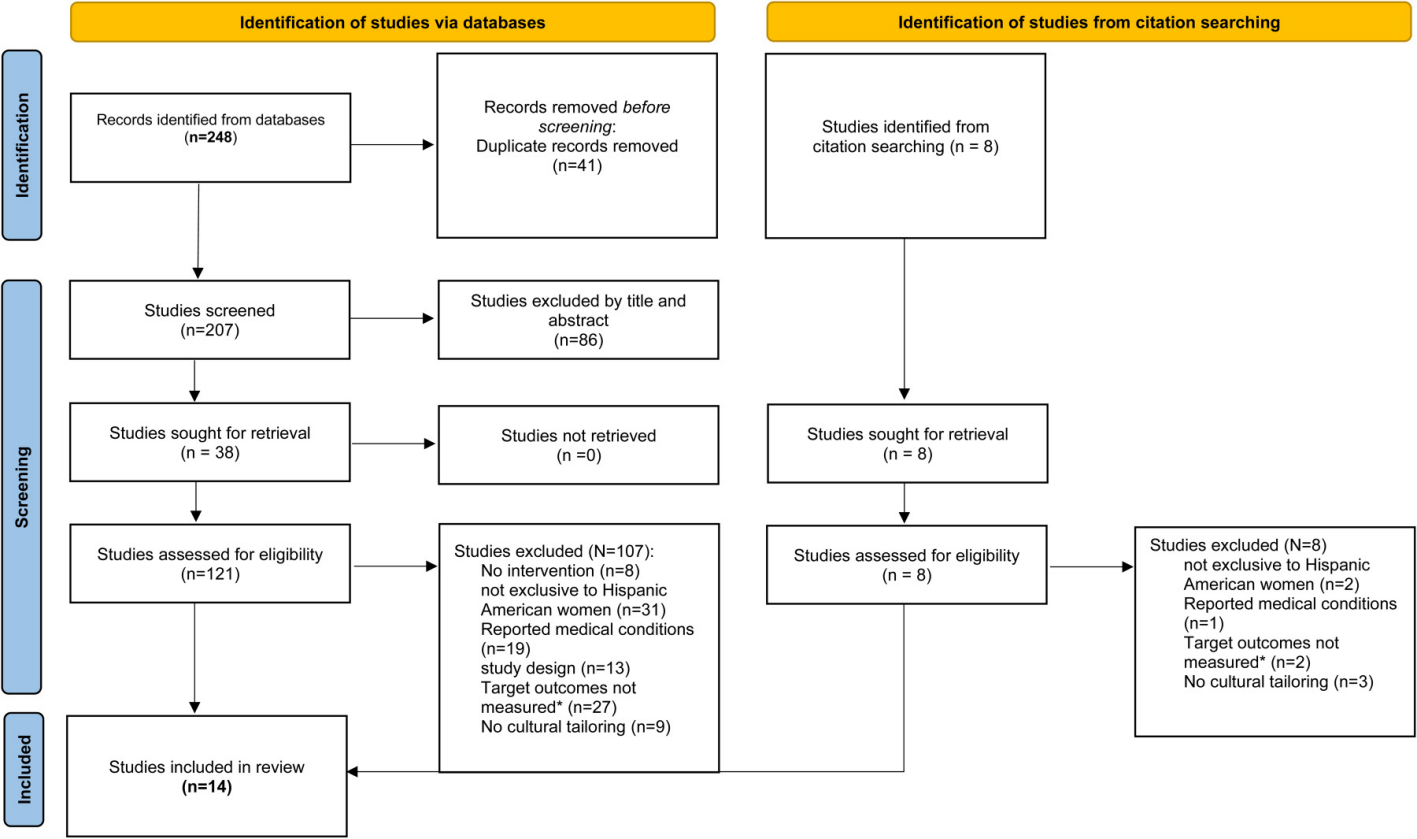
Search Flow Diagram Following PRISMA 2020 Guidelines. *Target Outcomes for This Review Are Reductions in Body Weight, BMI, or Waist Circumference.

The details of the screening and inclusion process are illustrated in a complete flow diagram in alignment with PRISMA 2020 principles (PRISMA, 2024) in Figure 1. During this process, the primary researcher identified 256 initial articles, removed 49 duplicate articles, and performed an initial screening of studies based on the title or abstract. This resulted in the removal of an additional 86 articles. From 121 full-text articles being assessed for eligibility, the following data points were then extracted: participant and population characteristics, study design and setting, intervention characteristics, and study outcomes. In this step, 107 articles were determined to be ineligible and excluded due to the following reasons: containing no intervention, or an intervention that did not utilize cultural tailoring; including the wrong population, or the population not being exclusively Hispanic American women; incorporating participants with reported medical conditions known to impact weight, participants reported to be taking medicines or supplements that can impact weight, or pregnant participants; employing an irrelevant study design; or not measuring our target outcomes.

### Study Characteristics

The final eligibility assessment resulted in a total of fourteen articles published between years 2007 and 2021, which were included in this systematic scoping review (Arredondo et al., 2017; Baldwin, 2015; Cherrington et al., 2015; Faucher & Mobley, 2010; Harralson et al., 2007; Hayashi et al., 2010; Keller & Cantue, 2008; Koniak-Griffin et al., 2015; Lindberg et al., 2012; Marquez & Wing, 2013; McCurley et al., 2017; O'Brien et al., 2015; Sanchez et al., 2021; Seguin et al., 2019). Eight of them were published within the last ten years, in 2014 or later (Arredondo et al., 2017; Baldwin, 2015; Cherrington et al., 2015; Koniak-Griffin et al., 2015; McCurley et al., 2017; O'Brien et al., 2015; Sanchez et al., 2021; Seguin et al., 2019). The characteristics of these studies are outlined above in Table 1. Of the fourteen articles, six were randomized controlled trials (Arredondo et al., 2017; Faucher & Mobley, 2010; Hayashi et al., 2010; Keller & Cantue, 2008; Koniak-Griffin et al., 2015; Marquez & Wing, 2013) and eight were quasi-experimental studies (Baldwin, 2015; Cherrington et al., 2015; Harralson et al., 2007; Lindberg et al., 2012; McCurley et al., 2017; O'Brien et al., 2015; Sanchez et al., 2021; Seguin et al., 2019). Nine were conducted as pilot studies to test methods and procedures (Baldwin, 2015; Cherrington et al., 2015; Faucher & Mobley, 2010; Lindberg et al., 2012; Marquez & Wing, 2013; McCurley et al., 2017; O'Brien et al., 2015; Sanchez et al., 2021; Seguin et al., 2019). Studies varied broadly in sample size (N = 15–1,093) and length (6 weeks to 2 years). Nine studies had considerably small sample sizes (Baldwin, 2015; Cherrington et al., 2015; Faucher & Mobley, 2010; Keller & Cantue, 2008; Lindberg et al., 2012; Marquez & Wing, 2013; O'Brien et al., 2015; Sanchez et al., 2021; Seguin et al., 2019), defined as less than 50 subjects (Fok et al., 2015). One study was 6 weeks (Sanchez et al., 2021), one 8 weeks (Cherrington et al., 2015), five 12 weeks (Baldwin, 2015; Harralson et al., 2007; Marquez & Wing, 2013; McCurley et al., 2017; Seguin et al., 2019) one 6 months (Koniak-Griffin et al., 2015), one 20 weeks (Faucher & Mobley, 2010), one 36 weeks (Keller & Cantue, 2008), three 12 months (Hayashi et al., 2010; Lindberg et al., 2012; O'Brien et al., 2015) and one 24 months in length (Arredondo et al., 2017).

To meet the criteria of an elevated weight status, nine studies recruited participants with a BMI exceeding 25 (Cherrington et al., 2015; Faucher & Mobley, 2010; Keller & Cantue, 2008; Koniak-Griffin et al., 2015; Lindberg et al., 2012; McCurley et al., 2017; O'Brien et al., 2015; Seguin et al., 2019), representing overweight or obesity weight status as an inclusion criterion. Five studies (Arredondo et al., 2017; Baldwin, 2015; Harralson et al., 2007; Hayashi et al., 2010; Sanchez et al., 2021) did not include weight status as a criterion to maintain minimal eligibility criteria with the purpose of increasing the number of participants. However, to be included in this review, studies lacking BMI criteria had to report a mean BMI of 30 or above for the sample, indicating obesity weight status.

Utilizing the EPHPP quality assessment tool for quantitative research, two studies were classified as “strong” (Arredondo et al., 2017; Koniak-Griffin et al., 2015), nine as “moderate” (Cherrington et al., 2015; Faucher & Mobley, 2010; Hayashi et al., 2010; Lindberg et al., 2012; Marquez & Wing, 2013; McCurley et al., 2017; O'Brien et al., 2015; Sanchez et al., 2021; Seguin et al., 2019), and three as “weak” (Baldwin, 2015; Harralson et al., 2007; Keller & Cantue, 2008) in quality as illustrated in Table 1. Receiving a “weak” final score resulted from having a high rate of dropouts or withdrawals, containing no mention of blinding, lack of adjusting for confounders, and/or lack of clarity about data collection methods. Consequently, as determinants of a weak-quality rating, these components were identified as key methodological areas of improvement across the three studies that received a poor rating. Despite the variation in quality, no notable trends were identified between ratings and study outcomes. However, it should be noted that in comparison with the rest of the studies, the two studies that scored “strong” were both longer studies, two years and six months in length respectively, and had larger sample sizes, 346 and 223 respectively (Arredondo et al., 2017; Koniak-Griffin et al., 2015). Both were *promotora*-led, had bilingual and bicultural staff, and offered participants materials created in a culturally sensitive manner. On the other hand, two of the three studies that received a “weak” rating had a smaller sample size of 18 and 48 (Baldwin, 2015; Keller & Cantue, 2008). Two studies also included translations from English-language materials (Baldwin, 2015; Harralson et al., 2007) rather than Spanish-language materials created specifically for Hispanic Americans taking into account key cultural nuances, which may be absent in direct translations.

Although only six studies utilized a randomized controlled design (Arredondo et al., 2017; Faucher & Mobley, 2010; Hayashi et al., 2010; Keller & Cantue, 2008; Koniak-Griffin et al., 2015; Marquez & Wing, 2013), which is broadly recognized as the ideal method of evaluating health behavior interventions and providing the most convincing evidence (Institute of Medicine Committee on Health Behavior: Research Practice Policy, 2001), all six randomized controlled trials reported similar results in terms of intervention effectiveness and producing weight loss among participants. However, it should be noted that only two of the trials received a “strong” quality rating (Arredondo et al., 2017; Koniak-Griffin et al., 2015), whereas three were rated as “moderate” (Faucher & Mobley, 2010; Hayashi et al., 2010; Marquez & Wing, 2013), and one was classified as “weak” (Keller & Cantue, 2008).

Consistent with commonly varying dropout rates in weight loss interventions (Sawamoto et al., 2016), the dropout rate ranges varied from 4% to 55% in this study. Seven studies reported a dropout rate of 20% or under (Arredondo et al., 2017; Cherrington et al., 2015; Koniak-Griffin et al., 2015; Marquez & Wing, 2013; McCurley et al., 2017; O'Brien et al., 2015; Sanchez et al., 2021), whereas four studies observed a dropout rate of 40% or over (Faucher & Mobley, 2010; Harralson et al., 2007; Keller & Cantue, 2008; Lindberg et al., 2012). The studies with the lowest dropout rates were all rated either “strong” or “moderate”, and those with the highest dropout rates received either a “moderate” or “weak” score.

After completion, six studies included a follow-up (Cherrington et al., 2015; Harralson et al., 2007; Koniak-Griffin et al., 2015; Marquez & Wing, 2013; McCurley et al., 2017; Seguin et al., 2019), while nine did not report any follow-up procedure (Arredondo et al., 2017; Baldwin, 2015; Faucher & Mobley, 2010; Hayashi et al., 2010; Keller & Cantue, 2008; Lindberg et al., 2012; O'Brien et al., 2015; Sanchez et al., 2021). Of the six studies that contained a follow-up, four reported significant weight loss (Cherrington et al., 2015; Koniak-Griffin et al., 2015; Marquez & Wing, 2013; Seguin et al., 2019). Three studies included follow-up measurements for weight loss, and all of them found that participants had lost additional weight at follow-up compared to post-intervention (Koniak-Griffin et al., 2015; Lindberg et al., 2012; Marquez & Wing, 2013). Collectively, these findings suggest that the implementation of a follow-up can potentially enhance observed weight loss outcomes in participants, particularly in studies with shorter durations and insignificant results upon intervention completion. Two follow-ups took place in form of post-intervention focus group discussions (Harralson et al., 2007; Seguin et al., 2019), which allowed researchers to further explore participants’ perceived barriers, facilitators, and motivators to weight loss in more detail, and to qualitatively assess the feasibility, accessibility, and effectiveness of the implemented intervention curriculums. The results of the focus group discussions are described later in this review.

### Intervention Characteristics

Detailed intervention characteristics including intervention type, implemented cultural tailoring strategies, delivery method and setting, measured target parameters, frequency of measurements, and intervention outcomes are highlighted in Table 2.

Of the fourteen interventions investigated in this review, seven included populations with an elevated weight status reflecting overweight or obesity, who were not recruited for being at-risk for any other particular health concern (Arredondo et al., 2017; Faucher & Mobley, 2010; Koniak-Griffin et al., 2015; Lindberg et al., 2012; Marquez & Wing, 2013; Sanchez et al., 2021; Seguin et al., 2019). Three interventions were designed for individuals at-risk for type 2 diabetes (Baldwin, 2015; McCurley et al., 2017; O'Brien et al., 2015), and four for individuals who were at-risk for cardiovascular disease (Baldwin, 2015; Harralson et al., 2007; Hayashi et al., 2010; Keller & Cantue, 2008), both due to an elevated weight status. Furthermore, four interventions were modeled after existing diabetes-focused prevention programs: three after the Diabetes Prevention Program (Cherrington et al., 2015; McCurley et al., 2017; O'Brien et al., 2015) and one after the National Diabetes Education Program (Baldwin, 2015). In these interventions, the primary purpose of seeking weight loss was to reduce participants’ risk status and prevent them from developing either type 2 diabetes or cardiovascular disease as weight loss is associated with improvements in both comorbidities (National Institute of Diabetes and Digestive and Kidney Diseases, 2023).

All interventions were delivered in-person in a community and group setting, utilizing a range of cultural tailoring strategies designed to facilitate weight loss among adult Hispanic American women, which is discussed in more detail under the following subheading. In all cases, the intervention curriculum contents focused on healthy eating and/or increasing physical activity (Arredondo et al., 2017; Baldwin, 2015; Cherrington et al., 2015; Faucher & Mobley, 2010; Harralson et al., 2007; Hayashi et al., 2010; Keller & Cantue, 2008; Koniak-Griffin et al., 2015; Lindberg et al., 2012; Marquez & Wing, 2013; McCurley et al., 2017; O'Brien et al., 2015; Sanchez et al., 2021; Seguin et al., 2019). Ten interventions combined dietary modification and physical activity (Baldwin, 2015; Cherrington et al., 2015; Hayashi et al., 2010; Koniak-Griffin et al., 2015; Marquez & Wing, 2013; McCurley et al., 2017; O'Brien et al., 2015; Sanchez et al., 2021; Seguin et al., 2019), one focused solely on dietary modification (Faucher & Mobley, 2010), and three solely in physical activity (Arredondo et al., 2017; Harralson et al., 2007; Keller & Cantue, 2008). Five interventions were designed to measure long-term (>6 months) outcomes (Arredondo et al., 2017; Hayashi et al., 2010; Koniak-Griffin et al., 2015; Lindberg et al., 2012; O'Brien et al., 2015), while nine measured outcomes in the short-term (<6 months) (Baldwin, 2015; Cherrington et al., 2015; Faucher & Mobley, 2010; Harralson et al., 2007; Keller & Cantue, 2008; Marquez & Wing, 2013; McCurley et al., 2017; Sanchez et al., 2021; Seguin et al., 2019).

All fourteen interventions investigated in this review were developed to test the impact of a behavioral lifestyle change curriculum measured in participants as a reduction of body weight, BMI, or waist circumference. Eight interventions examined the outcomes of body weight, BMI, and waist circumference (Arredondo et al., 2017; Baldwin, 2015; Harralson et al., 2007; Keller & Cantue, 2008; Koniak-Griffin et al., 2015; McCurley et al., 2017; O'Brien et al., 2015; Seguin et al., 2019), five measured body weight and BMI (Cherrington et al., 2015; Hayashi et al., 2010; Lindberg et al., 2012; Marquez & Wing, 2013; Sanchez et al., 2021), and one assessed body weight alone (Faucher & Mobley, 2010). The outcomes of each study are included in Table 2. Although excluded from the assessed target outcomes of this review, most interventions measured additional parameters that are commonly known to impact body weight (National Institute of Diabetes and Digestive and Kidney Diseases, 2023), including improvements in dietary intake and eating behaviors (Cherrington et al., 2015; Lindberg et al., 2012; Sanchez et al., 2021); increased physical activity or adherence to it (Arredondo et al., 2017; Baldwin, 2015; Cherrington et al., 2015; Faucher & Mobley, 2010; Hayashi et al., 2010; Koniak-Griffin et al., 2015; Lindberg et al., 2012; Sanchez et al., 2021; Seguin et al., 2019); level of fitness (Arredondo et al., 2017); body composition defined as body fat percentage (Keller & Cantue, 2008) or waist-to-hip ratio (Harralson et al., 2007); cardiorespiratory health (Seguin et al., 2019); cholesterol values (Hayashi et al., 2010); and blood pressure, glucose, and/or lipids (Baldwin, 2015; Cherrington et al., 2015; Harralson et al., 2007; Hayashi et al., 2010; Keller & Cantue, 2008; Koniak-Griffin et al., 2015; McCurley et al., 2017; O'Brien et al., 2015). Some studies also measured participants’ self-efficacy for weight loss, dietary practices, and/or physical activity (Marquez & Wing, 2013; Seguin et al., 2019); health knowledge (Harralson et al., 2007; Koniak-Griffin et al., 2015); self-rated health (Harralson et al., 2007); self-perceived weight (Sanchez et al., 2021); general health-related behaviors (Hayashi et al., 2010) and barriers to them (McCurley et al., 2017); family support (Marquez & Wing, 2013); stress levels (Harralson et al., 2007; McCurley et al., 2017); medical conditions (Arredondo et al., 2017; Harralson et al., 2007); and depressive symptoms (Cherrington et al., 2015; Harralson et al., 2007; McCurley et al., 2017). While these parameters were not analyzed as part of this study, they are highlighted for future research to explore as determinants and contributors to weight loss in the development of evidence-based interventions for the population, and to explore the topic from a holistic perspective including both physical and mental aspects.

### Development of Cultural Tailoring

The interventions included a mixture of culturally sensitive adaptations of previously conducted, larger-scale interventions or existing program curriculums (Baldwin, 2015; Cherrington et al., 2015; Harralson et al., 2007; Hayashi et al., 2010; Lindberg et al., 2012; McCurley et al., 2017; O'Brien et al., 2015; Sanchez et al., 2021), interventions designed in collaboration with a community advisory board consisting of community members and health experts focused on Hispanic populations (Cherrington et al., 2015; Koniak-Griffin et al., 2015; Marquez & Wing, 2013; Seguin et al., 2019), and staff-developed interventions (Arredondo et al., 2017). Two studies did not disclose how their interventions were developed (Faucher & Mobley, 2010; Keller & Cantue, 2008). Given the broad variation in intervention development strategies, no noteworthy trends between the strategies and reported outcomes could be established. However, it should be noted that two of the three studies rated as “weak” in quality were adaptations of existing curriculums (Baldwin, 2015; Harralson et al., 2007), and the third study did not disclose how it was developed (Keller & Cantue, 2008). On the other hand, one of the two studies that received a “strong” classification collaborated with a community advisory board (Koniak-Griffin et al., 2015), and the second one utilized a self-developed intervention (Arredondo et al., 2017). Studies rated as “moderate” were a blend of prior intervention adaptations (Cherrington et al., 2015; Hayashi et al., 2010; Lindberg et al., 2012; McCurley et al., 2017; O'Brien et al., 2015; Sanchez et al., 2021) and interventions developed in collaboration with a community advisory board (Cherrington et al., 2015; Marquez & Wing, 2013; Seguin et al., 2019), whereas one did not disclose how it was developed (Faucher & Mobley, 2010).

Developing culturally congruent weight loss interventions was detected to be more complex in promoting physical activity in culturally relevant ways than with dietary modification, with less feasible opportunities to do so. However, three interventions managed to innovatively encourage physical activity by incorporating Spanish-language music through offering Latin dancing (such as salsa dancing) as an exercise alternative (Harralson et al., 2007; Lindberg et al., 2012; Seguin et al., 2019).

### Delivery of Cultural Tailoring

Thirteen interventions were delivered by *promotoras,* or a similar concept of lay health workers who were bilingual and bicultural members of the targeted communities that they were trained to serve (Arredondo et al., 2017; Baldwin, 2015; Cherrington et al., 2015; Faucher & Mobley, 2010; Harralson et al., 2007; Hayashi et al., 2010; Keller & Cantue, 2008; Koniak-Griffin et al., 2015; Lindberg et al., 2012; Marquez & Wing, 2013; McCurley et al., 2017; O'Brien et al., 2015; Sanchez et al., 2021; Seguin et al., 2019). The delivery of interventions, health education, and assessments through the *promotora* model has been demonstrated to be effective among Hispanic women in prior health literature, especially in enhancing communication, which is widely recognized in health research as a challenge for Hispanic women residing in the U.S (O'Brien et al., 2015). The effectiveness of the *promotora* concept attributes to their capability of identifying with community norms, values, and culture that impact the health of Hispanic American communities, and their presence establishes credibility and trust among the population (O'Brien et al., 2015; Pekmezi et al., 2009). Health literature shows that *promotoras* are often regarded as community health leaders among Hispanic individuals (Arredondo et al., 2017). Given their unique role as facilitators of culturally tailored health information and agents of support in health promotion within their communities, they are even looked up to as inspirational role models (Arredondo et al., 2017).

Notably, in this review, the only intervention that was not delivered by *promotoras* or similar community health workers received a “weak” quality rating. However, given that only one intervention did not implement this method of delivery, no definite conclusions on its impact could be drawn.

### Cultural Tailoring of Intervention Components

While a degree of variation in cultural tailoring methods was observed across the interventions, all reviewed articles provided adequate descriptions of the strategies that were utilized. This greatly facilitated their assessment, which is featured in Table 2. Data from the reported intervention outcomes suggests that key determinants of culturally relevant and effective interventions were bicultural delivery with an alternative for a bilingual or Spanish-language intervention (Hayashi et al., 2010; Koniak-Griffin et al., 2015; Sanchez et al., 2021; Seguin et al., 2019), inclusion of bilingual and bicultural staff from the community (Arredondo et al., 2017; Cherrington et al., 2015; Faucher & Mobley, 2010; Harralson et al., 2007; Hayashi et al., 2010; Keller & Cantue, 2008; Koniak-Griffin et al., 2015; Lindberg et al., 2012; Marquez & Wing, 2013; McCurley et al., 2017; O'Brien et al., 2015; Sanchez et al., 2021; Seguin et al., 2019), translation and/or simplification of culturally adapted materials (Baldwin, 2015; Faucher & Mobley, 2010; Harralson et al., 2007; Lindberg et al., 2012; O'Brien et al., 2015; Sanchez et al., 2021; Seguin et al., 2019), incorporation of traditional Hispanic foods and recipes (Arredondo et al., 2017; Faucher & Mobley, 2010; Harralson et al., 2007; Keller & Cantue, 2008; Lindberg et al., 2012; Marquez & Wing, 2013; McCurley et al., 2017; Seguin et al., 2019), and peer participation for increased social support (Cherrington et al., 2015; Faucher & Mobley, 2010; Keller & Cantue, 2008; Lindberg et al., 2012; McCurley et al., 2017; O'Brien et al., 2015; Seguin et al., 2019). Implementing these elements encouraged intervention participation, increased participants’ engagement during the intervention, and removed culture-specific barriers to enhance weight loss outcomes.

### Outcomes of Cultural Tailoring

All fourteen culturally tailored interventions reviewed in this article were proven to be successful in producing weight loss among participants. Seven studies revealed statistically significant reductions in weight (Cherrington et al., 2015; Keller & Cantue, 2008; Koniak-Griffin et al., 2015; Lindberg et al., 2012; Marquez & Wing, 2013; O'Brien et al., 2015; Seguin et al., 2019), eight in BMI (Baldwin, 2015; Cherrington et al., 2015; Hayashi et al., 2010; Keller & Cantue, 2008; Koniak-Griffin et al., 2015; Lindberg et al., 2012; O'Brien et al., 2015; Seguin et al., 2019), and three in waist circumference (Koniak-Griffin et al., 2015; O'Brien et al., 2015; Seguin et al., 2019). Across studies, the mean reduction in body weight was −8.73 pounds (−3.96 kilograms), mean reduction in BMI units was −1.85, and mean reduction in waist circumference was −3.89 inches (−9.89 centimeters). Not only do these results confirm the substantial impact of culturally tailored strategies in treating overweight and obesity, but they also demonstrate the importance of access to approaches that are developed to meet the unique needs and preferences of Hispanic American women.

Of the studies that reported statistically significant weight loss, four reported exceptionally large reductions in body weight and BMI (Keller & Cantue, 2008; Lindberg et al., 2012; Marquez & Wing, 2013; O'Brien et al., 2015), calling for further exploration. These interventions were identified as most effective with a mean weight reduction of −16.32 pounds (−7.4 kilograms) and a mean BMI reduction of −3.91 units. The average intervention duration was 9 months. Three interventions consisted of dietary modifications and promoting physical activity (Lindberg et al., 2012; Marquez & Wing, 2013; O'Brien et al., 2015) and one focused on physical activity without a dietary component (Keller & Cantue, 2008). In addition to clinician-collected measurements to assess weight loss, three studies’ participants were required to self-monitor their dietary intake (Lindberg et al., 2012), adherence to physical activity (Keller & Cantue, 2008; Lindberg et al., 2012), self-efficacy for weight loss and exercise, and perceived social support (Marquez & Wing, 2013). Although the sample sizes of all four studies were below 50 (Keller & Cantue, 2008; Lindberg et al., 2012; Marquez & Wing, 2013; O'Brien et al., 2015), the findings highlight the significance of implementing longer study durations with well-rounded program components that promote comprehensive lifestyle changes through a healthy diet and physical activity. In addition, self-monitoring has been consistently established as a fundamental contributor to weight loss and intervention effectiveness in prior research examining their association (Burke et al., 2011), which aligns with the outcomes of these studies and provides grounds for the incorporation of self-monitoring in future program curriculums.

### Participant Feedback

Participant feedback collected through post-intervention follow-ups can be utilized to develop an in-depth understanding of the feasibility of different intervention components within the context of culturally tailored programs. In fact, similar results were observed in focus group discussions conducted as part of two studies following their interventions (Baldwin, 2015; Seguin et al., 2019). During these sessions, participants expressed finding a group setting motivating as a comfortable atmosphere with social support from other participants and staff, which made it easier for them to adhere to the lifestyle changes implemented during the intervention (Baldwin, 2015; Seguin et al., 2019). Community locations were found to be convenient, making the program more accessible and participants greatly appreciated culturally adjusted recipes, which they were able to share with their families when cooking meals at home (Seguin et al., 2019).

Consistent with prior literature, the primary perceived barrier to long-term lifestyle change adherence was lack of support or resistance toward change from family members and peers (Baldwin, 2015; Seguin et al., 2019). However, it was reported that over time, many families ended up supporting, and in some cases, even implementing the new lifestyle habits themselves as participants shared intervention learnings with them (Seguin et al., 2019). Other challenges included confusion about portion control, discouragement caused by weight fluctuations, and lack of free time preventing participants from adhering to healthy habits (Baldwin, 2015; Seguin et al., 2019). When requested to provide recommendations to improve the interventions, the participants of the first study's focus group suggested increasing the number of meetings per week, implementing longer exercise sessions, and extending the program duration to exceed 12 weeks (Seguin et al., 2019). The participants of the second study's focus group requested more content on advantageous nutrition and dietary changes to benefit weight loss with additional recipes and cooking tips, more general information on weight loss, and tools to manage stress, underlining these areas as most helpful in managing their weight during the intervention (Baldwin, 2015).

## Discussion

Of the studies included in this review, weight loss measured as reductions in body weight, BMI, and waist circumference was observed in all fourteen interventions. This evidences that culturally tailored lifestyle interventions are feasible, acceptable, and effective in producing weight loss and promoting healthy lifestyle practices among Hispanic American women. Culturally adjusted approaches to weight loss were well-received and perceived successful by the population. These strategies can contribute to the removal of common barriers to weight loss in Hispanic American women while facilitating their interest and adherence to weight management interventions.

Successful development of targeted weight loss programs requires a broad understanding of the effectiveness of culturally tailored approaches and adjusted interventions. Across the reviewed studies, key intervention components consisted of the inclusion of a bilingual and bicultural staff, offering the intervention in English and Spanish, translating and simplifying materials, adjusting interventions with culturally relevant foods and recipes, and encouraging the participation of peers. Creating materials intended for the population, or modifying existing materials appeared to be more successful than simply translating materials from original English-language versions. These findings were reflected in a comparable fashion during post-intervention focus group discussions, further strengthening the post-intervention observations. Four studies achieved the most impactful weight loss outcomes with a mean body weight reduction exceeding 15 pounds, and a mean BMI reduction of nearly 4 units. These interventions had an average duration of 9 months, three included an intervention with a dietary and physical activity component, and all four required participants to self-monitor health-related behaviors. Understandably, shorter interventions resulted in less meaningful results given the evident time constraints. The researchers of the shortest study with a 6-week intervention reported “not expecting” statistically significant changes in participants’ weight nor BMI given the duration (Sanchez et al., 2021), which emphasizes the importance of setting an adequate length during the planning stages of an intervention.

The findings of this review are consistent with prior literature examining the influence of culturally tailored approaches in behavioral health interventions. Although there are no similar studies assessing the effectiveness of culturally tailored weight loss interventions in Hispanic American women, when compared to existing literature on interventions designed for other ethnic or racial groups, it appears that Hispanic American women may benefit more from them than others. For example, a systematic review exploring the effectiveness of such interventions in African American women, 60% of the studies implementing cultural tailoring produced significant weight change in participants (Kong et al., 2014). One potential explanation for the success among Hispanic Americans may be the language barrier that does not exist for other large ethnic or racial groups in the U.S., making cultural tailoring a key component in weight loss programs geared toward the Hispanic population. Existing research has suggested that language, socioeconomics, and lack of cultural relevance are among the most critical barriers preventing Hispanic populations from benefitting from pre-existing health programs designed for the English-speaking population (Pekmezi et al., 2009). Despite the wide recognition that treatments are more effective when they are adapted to the specific needs of a population (Lindberg et al., 2013) and that the purpose of implementing culturally tailored strategies is to eliminate existing barriers, the number of weight loss interventions targeting Hispanic American women remains scarce. As such, the findings of this study fill an important gap in research, contributing to the limited knowledge on the impact of cultural tailoring and weight loss efforts among the population.

## Limitations

Limitations of this research include variation among sample sizes, intervention length and content, cultural tailoring strategies, and reporting of outcome measures. The inclusion of multiple pilot studies results in some of the included articles having small sample sizes and short durations. Collectively, these limitations can result in challenges to conduct future meta-analyses on the topic. As described earlier, certain limitations in research quality were observed with three studies receiving a “weak” quality rating.

Another limitation is the lack of clinical studies comparing culturally tailored interventions to non-tailored interventions in Hispanic American women. If such studies existed, it would allow us to conduct a more accurate analysis on the effectiveness of cultural tailoring among the population. In the absence of intervention comparisons, few published articles are available on cultural tailoring in weight loss interventions targeting Hispanic American women overall. Consistent with other areas of research focusing on the Hispanic population living in the U.S., studies have traditionally fallen short of including individuals with varying ethnic backgrounds and identities. Research on Hispanic Americans primarily includes individuals of Mexican descent, which can pose an additional limitation. In this review, seven studies reported that at least 70% of the sample was comprised of Mexican American women (Cherrington et al., 2015; Faucher & Mobley, 2010; Keller & Cantue, 2008; Koniak-Griffin et al., 2015; Lindberg et al., 2012; McCurley et al., 2017; Sanchez et al., 2021). As a highly heterogenous population, Hispanic individuals differ in ethnic backgrounds and identities, genetics, geographic locations, dietary preferences, culture, language, traditions, and levels of acculturation (Alemán et al., 2023; Lindberg et al., 2013). These are important aspects to consider in the development of culturally tailored interventions to maximize their outcomes in Hispanic American women with diverse backgrounds (Alemán et al., 2023).

Additionally, many of the reviewed studies investigated our target outcomes as secondary outcomes. This is suboptimal as it may imply a risk of inconsistencies in measured outcomes and data collection methods. In some cases, although our target weight loss outcomes were described to be among the measured parameters, their reporting was vague or incomplete. As an example, certain studies left p-values of statistical significance unreported for some of the parameters, limiting our ability to conduct a thorough assessment of all measured outcomes. While many studies reported weight loss outcomes in a comprehensive manner, some left out important details such as the percentage of lost weight across the sample from baseline to post-intervention, or failed to disclose relevant data that would have allowed us to calculate it. As losing 5–10% of body weight is generally regarded as clinically significant (Ryan & Yockey, 2017), having access to this data would have permitted us to determine the clinical significance of the mean weight loss observed among each study's sample.

As a suggestion for minimizing variation in outcome reporting in future research, when a culturally sensitive approach is implemented in an intervention, its effectiveness should be measured by examining both participant acceptance and measures of association between the tailoring strategies and the outcomes such as changes in body weight, BMI, or waist circumference as primary parameters of weight loss. Studies comparing culturally tailored interventions with non-culturally tailored interventions using a control group are urgently needed.

## Conclusion

This is the first systematic scoping review to rigorously summarize culturally appropriate strategies to promote lifestyle modification for weight loss among Hispanic American women with overweight or obesity, and to synthesize the outcomes of these approaches. Expanding upon the current understanding of the disproportionate burden that obesity poses for the population, this study contributes to modern research by analyzing key intervention characteristics and outcomes, including a comprehensive exploration of strategic aspects of cultural tailoring, which have not been previously interpreted in weight management studies targeting this uniquely at-risk yet understudied group.

Based on the findings of this review and previously discussed data on poor dietary habits and inactivity rates among the population compared to other groups, Hispanic individuals in the U.S. face an evident need for tailored programs that are designed to take their preferences and needs into account, arguably even more so than other groups. The findings of this review corroborate that culturally adjusted strategies delivered within participants’ own communities should be used to develop and implement relevant community programs and initiatives for the prevention and management of obesity as a public health priority.

In addition, these initiatives are suggested to consider women's pivotal influence and role in Hispanic families and communities. Depending on the level of acculturation, women are expected to be in charge of cooking and providing meals for the rest of the family in many Hispanic cultures and traditions (Sprague Martinez et al., 2022). Not only does it place them in a unique position to improve their own health outcomes, but those of their families, communities, and future Hispanic American generations.

**Figure 2.**
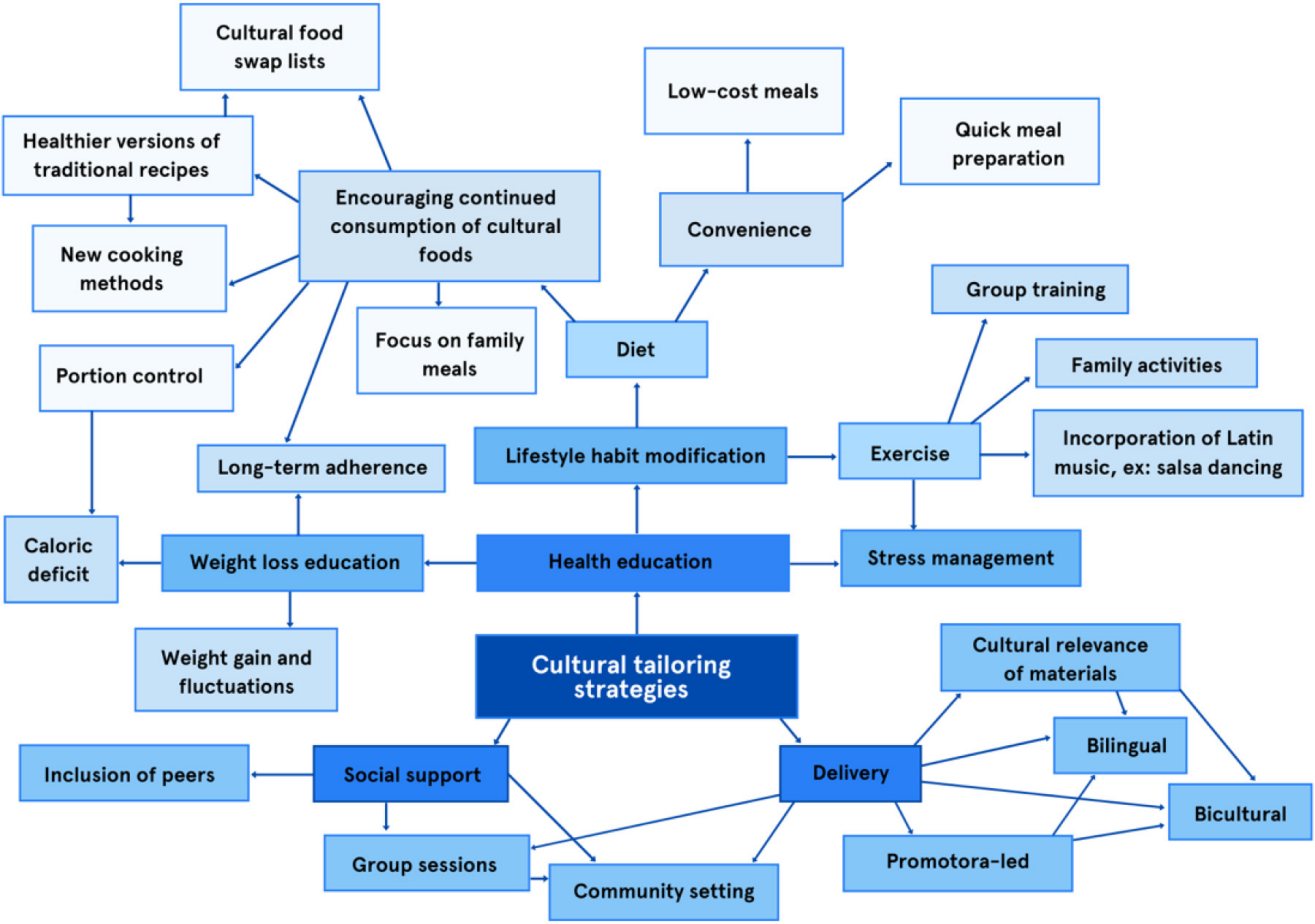
Key Cultural Tailoring Elements and Proposed Techniques to Guide Future Community Programs and Research.

This review provides rationale for further research to build upon the examined intervention approaches. Based on the findings of this review, Figure 2 is offered to summarize key elements of cultural tailoring strategies extracted from the reviewed studies, along with proposing innovative techniques to be implemented in the future. Its purpose is to facilitate the development of new community initiative curriculums, and to guide further research to suit diverse Hispanic backgrounds and varying levels of acculturation.

While the findings of this review demonstrate that there is robust evidence to support and advocate for the effectiveness of culturally tailored weight loss interventions, there is simultaneously an acute need for more rigorous clinical trials. Strong designs, larger sample sizes, intervention durations exceeding six months, and qualitative post-intervention follow-ups are required to comprehensively address the long-term effectiveness of culturally tailored interventions from a holistic perspective including both physical and mental aspects. Congruently and adequately designed interventions can support the development of effective weight management systems and contribute to improved health outcomes among this highly vulnerable population.

## Supplemental Material

sj-docx-1-hci-10.1177_15404153251383131 - Supplemental material for The Impact of Culture in Weight Loss Intervention Effectiveness Among Hispanic American Women with Overweight or Obesity: A Systematic Scoping Review Supplemental material, sj-docx-1-hci-10.1177_15404153251383131 for The Impact of Culture in Weight Loss Intervention Effectiveness Among Hispanic American Women with Overweight or Obesity: A Systematic Scoping Review by Anna V. Garcia, MSc, Elizabeth Falls, PhD, Elizabeth Dodge, PhD, RD, and Basil H. Aboul-Enein, EdD, MSc, MPH, MA in Hispanic Health Care International
