# Supplementary material for: The Impact of Culture in Weight Loss Intervention Effectiveness Among Hispanic American Women with Overweight or Obesity: A Systematic Scoping Review
Source: Hisp Health Care Int. 2025 Oct 17;24(3):153–71. doi: 10.1177/15404153251383131 (PMC13443355; doi:10.1177/15404153251383131)
Supplement: Supplementary file 1 [file sj-docx-1-hci-10.1177_15404153251383131.docx]

**SUPPLEMENTARY MATERIAL**

"Hispanic American" [All Fields]; "Hispanic Americans" [All Fields]; “Hispanic” [All Fields]; “Hispanics” [All Fields]; “Latina” [All Fields]; “Latinas” [All Fields]; “Latin” [All Fields]; “Latin American” [All Fields]; “Spanish-speaking” AND "Women” [All Fields]; "Female" [All Fields] AND “Overweight” [All Fields]; “Obesity” [All Fields]; “Obese” [All Fields] AND “Weight Loss” [All Fields]; “Weight” [All Fields]; “Weight Management” [All Fields]; “Weight Reduction” [All Fields]; “Body Weight” [All Fields]; “Body Mass Index” [All Fields]; “BMI” [All Fields]; “Waist Circumference” [All Fields] AND “Intervention” [All Fields]; “Program” [All Fields]; “Lifestyle Intervention” [All Fields], “Lifestyle” [All Fields]; “Losing Weight” [All Fields]; “Health Promotion” [All Fields]; “Diet” [All Fields]; “Calorie Restriction” [All Fields]; “Caloric Restriction” [All Fields]; “Exercise” [All Fields]; “Exercising” [All Fields]; “Physical Activity” [All Fields]; “Fitness” [All Fields]
